# Negative Appraisals of the COVID-19 Social Impact Associated With the Improvement of Depression and Anxiety in Patients After COVID-19 Recovery

**DOI:** 10.3389/fpsyt.2021.585537

**Published:** 2021-04-15

**Authors:** Wentao Chen, Yumeng Ju, Bangshan Liu, Mei Huang, Aiping Yang, Yun Zhou, Mi Wang, Mei Liao, Kongliang Shu, Jiyang Liu, Yan Zhang

**Affiliations:** ^1^Department of Psychiatry, The Second Xiangya Hospital, Central South University, Changsha, China; ^2^Hunan Key Laboratory of Psychiatry and Mental Health, China National Clinical Research Center on Mental Disorders (Xiangya), China National Technology Institute on Mental Disorders, Hunan Technology Institute of Psychiatry, Mental Health Institute, Central South University, Changsha, China; ^3^Department of Obstetrics & Gynecology, The First Hospital of Changsha, Changsha, China; ^4^Department of Orthopedics, The First Hospital of Changsha, Changsha, China; ^5^Department of Neurology, The First Hospital of Changsha, Changsha, China; ^6^Administrative Office, The First Hospital of Changsha, Changsha, China

**Keywords:** coronavirus disease 2019, patients, mental health, psychology, depression, anxiety, negative appraisals

## Abstract

**Objective:** Little is known about the factors affecting the recovery of mental health in COVID-19 patients. The purpose of this study is to look into the change of psychological distress and to explore the role of negative appraisals in the improvement of psychological distress in COVID-19 patients after they recovered from the infection.

**Methods:** We conducted a longitudinal survey on patients with COVID-19 infection in Changsha. The 9-item Patient Health scale, the 7-item Generalized Anxiety Disorder scale, and a newly developed measure, the COVID-19 Impact Scale (CIS) were applied to assess patients' depression, anxiety, and negative appraisal toward COVID-19 infection during their hospitalization and 1 month post-discharge.

**Results:** Seventy-two patients were included in the analysis. A significant decrease in anxiety and depression levels was observed after patients were discharged from hospital. Two meaningful factors of the CIS were extracted based on factor analysis, namely “health impact,” and “social impact.” The change of social impact explained the 12.7 and 10.5% variance in the depression and anxiety symptom improvement, respectively.

**Conclusions:** Change in negative appraisals, especially the appraisals related to COVID-19 social impact may play a vital role in the relief of psychological distress of infected patients. Therefore, a cognitive and social care perspective might be considered when promoting the mental health recovery and readjustment to society among COVID-19 patients.

## Introduction

Since the end of 2019, coronavirus disease 2019 (COVID-19) has emerged from China and spread rapidly to other countries. As of February 2021, the virus has been transmitted to 213 countries, had more than 100 million confirmed cases, and a death toll of 2.2 million has been reached (1). The highly contagious disease has had significant negative impacts on people's health, both physically and mentally.

Previous studies on viral infectious diseases, such as Severe Acute Respiratory Syndrome (SARS) and Ebola virus disease (EVD), have shown that emerging infectious disease can cause serious psychological distress (i.e., depression and anxiety) in patients during the acute phase of the disease (2–4). Studies also showed that the infection may have long-term effects on the mental health of the survivors (5–7). One study pointed out that 1 year after the outbreak of SARS, a large portion of survivors were still at a high level of stress, with more than 64% of patients developing mental disorders (8). One study investigated neurological and neuropsychiatric complications in patients with COVID-19 and found that 23 of 123 patients showed altered mental health status that fulfilled psychiatric diagnosis (9). Another qualitative study provided evidence that patients with COVID-19 experienced mental distress including anxiety and fear during the early stages of the disease (10). Moreover, a rapid review indicated that COVID-19 infection adversely affects the mental health status in patients (11). These findings suggest that we need to pay attention not only to the physical recovery of the infected patients, but also to the mental recovery of patients in the current COVID-19 epidemic.

Several factors were suggested to play an important role in the mental health status of the infected patients, including the disease condition (12) or being isolated or quarantined during hospitalization (13). However, these short-term influencing factors do not sufficiently explain the continued mental distress in patients after they have recovered from the infection. There are other factors that influence the long-term mental health condition of patients.

Negative appraisals were found to play an important role in the course of mental distress and its prognosis (14–17). A study of collegiate students in China showed that COVID-19-related negative appraisals was positively correlated with their emotional distress (18). A previous SARS study on medical staff reported a high level of post-traumatic symptoms among those who had perceived discrimination or felt rejected (19). In addition, another SARS study revealed a significant correlation between negative appraisals of the disease and symptoms of depression and anxiety (20). These lead us to the hypothesis that negative appraisals of the COVID-19 infection may exert influence on the mental recovery of patients.

In light of the above, we conducted a longitudinal study involving patients with COVID-19 in Changsha. The purpose of this study is as follows: first, to track the change in depression and anxiety levels in patients before and after they have recovered from the COVID-19 infection. Second, to explore the role of negative appraisals in depression and anxiety symptom improvement in patients after COVID-19 recovery.

## Methods

### Participants

This study was approved by the ethics review boards of The First Hospital of Changsha. The target population comprised all the COVID-19 patients admitted to The First Hospital of Changsha (Changsha, Hunan, China), which is the only designated hospital in Changsha city to receive patients with COVID-19 for treatment.

We conducted a longitudinal questionnaire survey on all hospitalized patients with COVID-19 from February to April 2020. COVID-19 was diagnosed according to the diagnosis and treatment plan for COVID-2019 released by the National Health Commission (21). Patients who consented to participate and were able to complete the online survey were included in the study. Patients who were below the age of 18, were unable to provide informed consent for any reason, or could not use mobile devices to complete all the questionnaires on their own were excluded from the study.

### Study Design

A baseline survey was conducted during patients' hospitalization. The nurse in the isolation ward informed the eligible patients that the online survey was set up to investigate the prevalence and related factors of mental distress in the COVID-19 pandemic. After acquiring the verbal consent of the participants, the nurse provided the patients with the website to fill out the questionnaire. The patients signed the electronic informed consent on the homepage of the website before the survey. At the end of the questionnaire, participants were asked about their needs for mental health services.

A follow-up survey was conducted 1 month after the patients were discharged from the hospital. The researchers contacted the patients by phone to learn about their current physical and mental health status. After obtaining verbal consent, the researchers sent patients a website link of the follow-up survey. Participants were asked to sign the electronic informed consent before they completed the questionnaire. Furthermore, mental health services were provided according to their self-reported mental health service needs after the follow-up survey, and patients who met the cut-off points (10 points) for PHQ-9 or GAD-7 were suggested for further individual evaluation and psychological counseling.

### Study Measures

The patients who agreed to participate in the study were asked to fill out a questionnaire recording their demographic information, clinical characteristics, and psychological variables. The demographics included age, gender, marital status, and years of education. The clinical characteristics comprised past medical history, duration of hospitalization, and the severity of COVID-19. The Chinese version of the 9-item Patient Health Questionnaire (PHQ-9; range, 0–27) and the 7-item Generalized Anxiety Disorder scale (GAD-7; range, 0–21) were used to assess the severity of the patients' depression and anxiety symptoms. The Chinese version of PHQ-9 and GAD-7 showed good reliability and validity in general hospital inpatients (22, 23). The COVID-19 disease-related questionnaire was used to reflect the degree of the patients' negative attitudes toward the infection. This 9-item scale was referred to the self-compiled questionnaire of the SARS Impact Scale (20). These nine items assessed the most common negative appraisals during and after the acute phase of coronavirus infection. A Likert scale was used to compile the scale and each question had five options with a score of 1–5 (1 = “not worried at all” and 5 = “extremely worried”). We named the revised self-rating scale COVID-19 Impact Scale (CIS). Moreover, we calculated the change scores of PHQ-9 /GAD-7/CIS by subtracting the baseline scores from the follow-up scores to indicate the improvement of mental distress or the change of negative appraisals. Other variables included patients' isolation sites during the first 2 weeks after getting discharged (categorized as at home or at the designated hotel) and mental health service utilization throughout the follow-up period (categorized as received or not received online or telephone supportive counseling).

### Statistical Analysis

All analyses were conducted in SPSS Version 25.0. The significance evaluation was set at *P* < 0.05 (two-tailed). For continuous variables, a Kolmogorov-Smirnov test was carried out to test for normality of the distribution. Data with normal distribution were presented as mean and standard deviation, and an independent sample *t*-test was used to compare the differences between the patients included and excluded. Data that were non-normally distributed were presented as median and quartile distance, and a Mann–Whitney *U* test was used for comparison of the two groups. Categorical variables were presented as frequency and percentages in each category, and a Pearson's chi-squared test was used to detect differences between the included and excluded patients as well.

Exploratory factor analysis using principal component analysis (PCA) and Varimax rotation was conducted to explore the underlying structure of CIS as well as summarize the variables of CIS. The appropriateness of factor analysis was assessed with the Bartlett's test of sphericity and the Kaiser-Meyer-Olkin (KMO) measure. The minimum factor loading cut-off point was set at 0.4 and factors with eigenvalues > 1 were selected. The internal consistency of each factor was assessed by Cronbach's α. Items were combined if Cronbach's α reached 0.80.

Repeated measure of the General Linear Model (GLM) was used to examine the effect of time on depression and anxiety as well as on patients' negative attitudes toward the infection. Demographics (age, gender, education, marriage), clinical characteristics (past medical history, severity of COVID-19, and duration of hospitalization), and supportive counseling during isolation (received or not) were included in the GLM as covariates, but were finally removed from the model as none of them showed significant effect.

Pearson correlation analysis was applied to explore the variables (i.e., demographic variable, clinical variable, and negative appraisals) that were associated with depression and anxiety symptom improvement (baseline scores—follow-up scores). Linear regression analyses were used to examine variables that explained the variance of patients' depression and anxiety symptom improvement. Variables significantly correlated with depression and anxiety symptom improvement were used as independent variables in the linear regression analysis, and depression and anxiety symptom improvement served as dependent variables, respectively.

## Results

### Participants

A total of 251 patients with COVID-19 were admitted to the hospital during the peak stage of COVID-19 in China. By February 9, 39 patients had recovered and been discharged from hospital. Besides, six subjects under 14 years old were excluded from our study. Questionnaires were sent to 206 patients in hospitalization and 163 patients gave their consent to take part in the study at baseline. The overall response rate at baseline was 79.13%. During follow-up, 18 patients dropped out because of incorrect phone numbers or missed calls. Among 145 potential participants who answered the calls, 39 subjects did not consent to participate in the follow-up survey at the time of informed consent. The remaining 114 replies represented a response rate of 78.6%.

There were 42 patients who had not fully completed the Coronavirus Impact Scale (CIS) during baseline or the follow-up time point. Eventually, 72 patients who filled in the CIS in both time points were included in the analysis. Table 1 shows the demographic and clinical characteristics of the patients. The median age was 38 years (IQR, 29–47) among the included patients. Gender was closely balanced between men and women with 37 male patients (57.1%). Twenty-three patients (31.9%) had a history of physical disease and three patients (4.2%) had a history of mental disorder. The average length of stay was 17.7 days. There were no significant differences between included and excluded (the participants dropped out or with incomplete data) patients in the demographics, clinical characteristics, and psychological dimension.

**Table 1:** Demographics and clinical characteristics of patients.

**Demographics and clinical characteristics**	**Patients at baseline** **(*n* = 163)**	**Patients included** **(*n* = 72)**	**Patients excluded** **(*n* = 91)**	***t***	***P***
Age, median (IQR)	40 (31–50)	38 (29–47)	41 (31–50)	0.178	0.859^a^
Gender, *n* (%)				0.275	0.600^b^
Males	80 (49.1)	37 (51.4)	43 (47.3)		
Female	83 (50.9)	35 (48.6)	48 (52.7)		
Marriage, *n* (%)				0.082	0.775^b^
Unmarried or divorced	39 (23.9)	18 (25.0)	21 (23.1)		
Married	124 (76.1)	54 (75.0)	70 (76.9)		
Education, *n* (%)				1.312	0.252^b^
High school and below	60 (36.8)	2 3(31.9)	37 (40.7)		
Undergraduate	86 (52.8)	40 (55.6)	46 (50.5)		
Postgraduate and above	17 (10.4)	9 (12.5)	8 (8.8)		
History of physical disease, *n* (%)	25 (15.3)	23 (31.9)	22 (24.2)	0.972	0.332^b^
History of mental disorder, *n* (%)	6 (3.7)	3 (4.2)	3 (3.3)	−0.663	0.509^b^
Duration of hospitalization, mean ± SD	18.2 ± 8.3	17.7 ± 8.6	18.5 ± 8.0	0.629	0.530^c^
Severity of COVID-19, *n* (%)				0.058	0.810^b^
Severe	19 (11.7)	9 (12.5)	10 (10.9)		
Mild	144 (88.3)	63 (87.5)	81 (89.1)		
Isolation site				N/A	N/A
Home	N/A	33 (45.8)	N/A		
Designated hotel	N/A	39 (54.2)	N/A		
Mental health service utilization, *n* (%)				N/A	N/A
Received	N/A	34 (47.2)	N/A		
Not received	N/A	38 (52.7)	N/A		
PHQ-9, mean ± SD	5.25 ± 5.53	5.70 ± 5.52	4.90 ± 5.55	−0.917	0.360^c^
Median, range	4 (1–7)	4 (2–7)	3 (1–6)		
GAD-7, mean ± SD	4.85 ± 5.25	5.59 ± 5.70	4.28 ± 4.83	−1.587	0.115^c^
Median, range	4 (0–7)	5 (0–7)	3 (0–7)		

*n, number of participants; IQR, interquartile range; N/A, not available; SD, standard deviation*.

a P-values obtained by Mann–Whitney U test;

b P-values obtained by Pearson's chi-square test;

c *P-values obtained by student t test*.

### Psychological Distress in Patients

**Figure 1:**
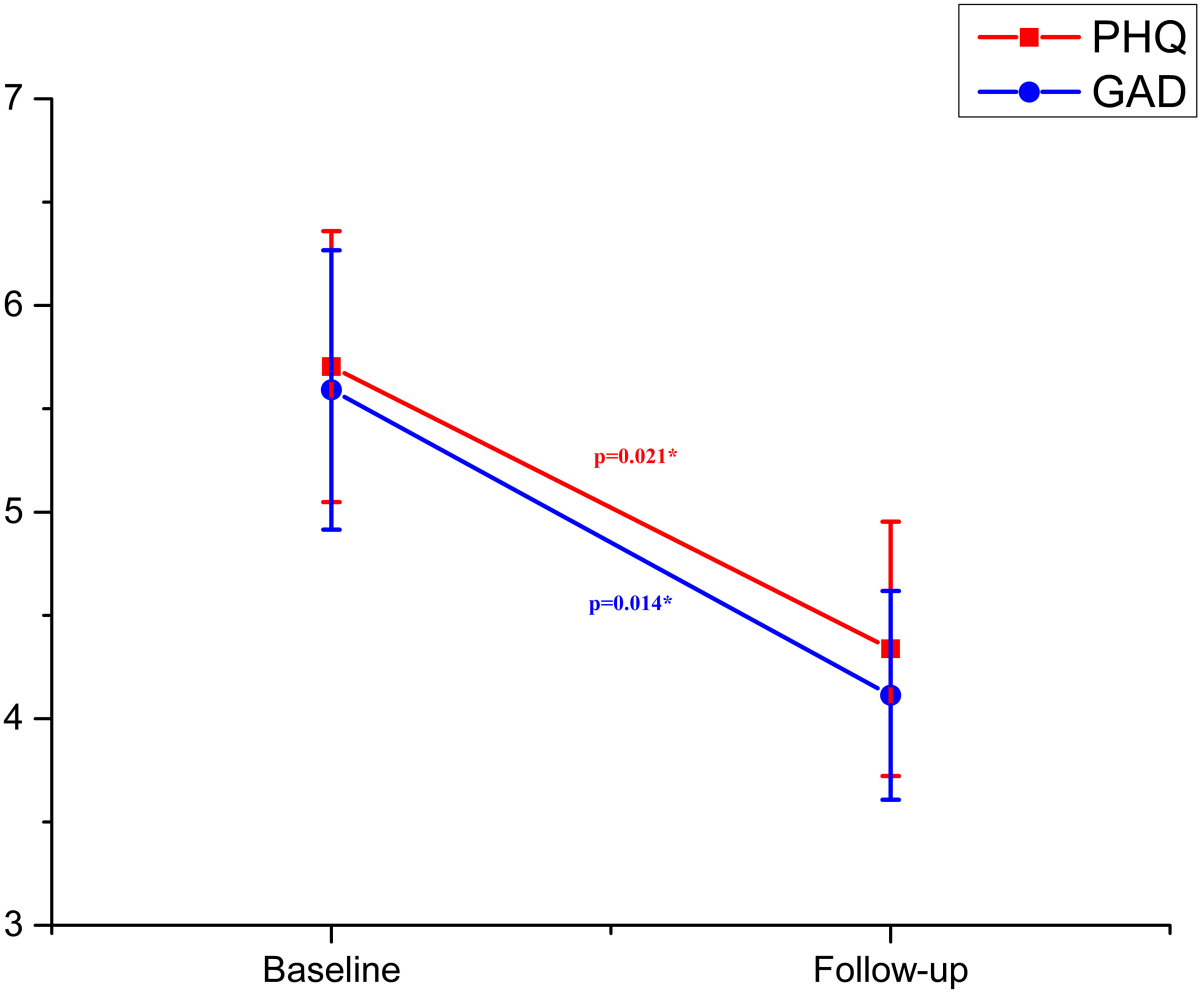
Depression and anxiety in patients with coronavirus infection at baseline and follow-up (error bars indicate standard errors of the variables). *P < 0.05.

Depression and anxiety scores during baseline and follow-up are shown in Table 2. The median scores of PHQ-9 and GAD-7 at baseline were 4 (IQR, 2–7), and 5 (IQR, 0–7), respectively. A total of 42.3% of the patients reported at least mild depression and 50.7% of patients reported anxiety symptoms. One month after discharge, the scores of both scales decreased (PHQ: 3, IQR 0–7; GAD: 3, IQR 0–7). GLM analysis revealed a significant effect of time on depression levels (*F* = 5.593, *P* = 0.021; Figure 1) and anxiety levels (*F* = 6.387, *P* = 0.014; Figure 1).

**Table 2:** Depression and anxiety in COVID-19 patients.

	**Baseline**	**Follow-up**
**PHQ-9**		
Mean,SD	5.70 ± 5.52	4.43 ± 5.19
Median, range	4 (2–7)	3 (0–7)
**GAD-7**		
Mean, SD	5.59 ± 5.70	4.11 ± 4.26
Median, range	5 (0–7)	3 (0–7)

*PHQ-9, 9-item Patient Health Questionnaire; GAD-7, 7-item Generalized Anxiety Disorder scale; n, number of participants; SD, standard deviation*.

### Negative Appraisals in Patients

The Bartlett's test of spherical (χ^2^ = 371.98, df =36, *P* < 0.001) and KMO measure (KMO = 0.845) showed that the CIS was suitable for factor analysis. The internal consistency of the total score was 0.932. PCA analysis yielded two factors, explaining 69.84% of the variance of CIS data. The two factors were named as health impact and social impact, respectively (Table 3). Both factors showed good internal consistency; Cronbach's alpha reached 0.896 for health impact and 0.885 for social impact. Therefore, the subsequent analysis will use the total scores of the two dimensions.

**Table 3:** Results of the exploratory factor analysis of CIS.

	**Patients with COVID-19**	
Kaiser-Meyer-Olkin measure	0.845	
Bartlett's test	Chi-square = 371.977, *P* < 0.000	
Component	Healthy impact	Social impact
Eigenvalue	5.13	1.16
Percentage of variance	57.00	12.84
Rotated component matrix, items (loading)	1. The medicine will have side effects on me (such as memory loss, etc.). (0.75) 2. I'm worried about passing the virus on to my family. (0.79) 3. COVID-19 will permanently damage my health. (0.83) 4. Even if I recover, I could be re-infected. (0.77) 5. I will have a mental problem. (0.63) 6. I will become a virus carrier. (0.76)	7. I will lose my job/have financial problems in the future. (0.84) 8. People will discriminate against me because I had COVID-19. (0.77) 9. My family will be destroyed by COVID-19. (0.87)

**Figure 2:**
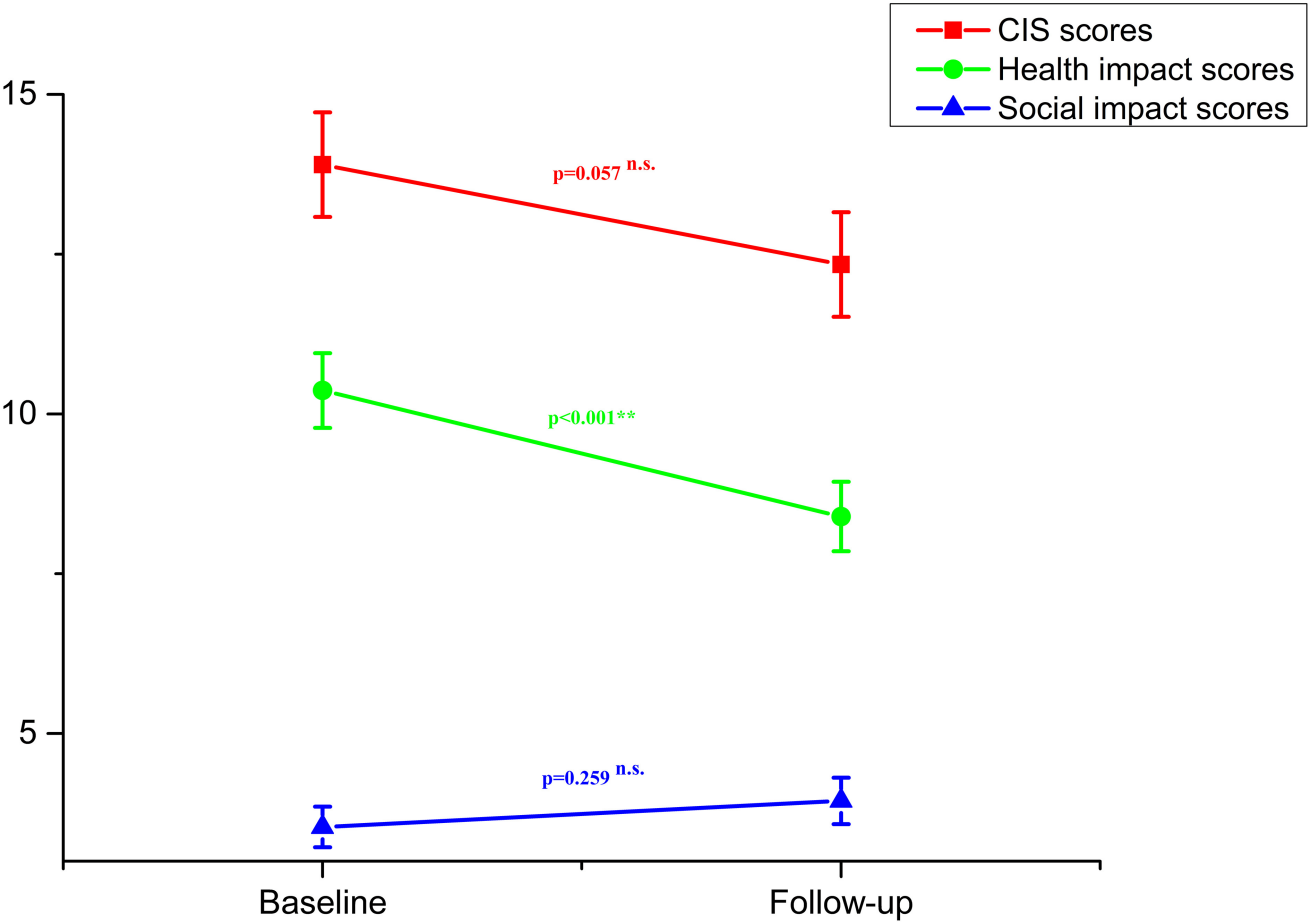
Total COVID-19 Impact Scale (CIS) scores, scores of health impact and scores of social impact in patients with coronavirus infection at baseline and follow-up (error bars indicate standard errors of the variables). n.s, non-significant; **P < 0.01.

No significant effect of time was detected by the GLM analysis on the total scores of the CIS (*F* = 3.75, *P* = 0.057; Figure 2). However, a significant effect of time on the health impact scores was observed (*F* = 11.94, *P* < 0.001; Figure 2). Particularly, the health impact scores decreased over time. Besides, there was no significant effect of time on the social impact scores (*F* = 1.29, *P* = 0.259; Figure 2).

### Relationship Between the Change of Negative Appraisals and Symptom Improvement in Depression and Anxiety

To explore the variables associated with the depression and anxiety symptom improvement, correlation analyses were conducted. Pearson correlation analysis showed that the patients' demographic data, clinical characteristics, or negative appraisals (total score or factor score) had no significant correlation with the improvement in PHQ-9 scores and GAD-7 scores. However, the change of social impact was significantly correlated with the improvement in PHQ-9 scores and GAD-7 scores (PHQ: *t* = 0.440, *P* < 0.001; GAD: *t* = 0.251, *P* = 0.038). The change of health impact was significantly correlated with the improvement in PHQ-9 scores (*t* = 0.29, *P* = 0.017), but not with the improvement in GAD-7 scores (Table 4).

**Table 4:** Association between the change of negative appraisals and patients' depression and anxiety symptom improvement.

	**Change of** **PHQ-9**	**Change of** **GAD-7**	**Change of** **health impact**	**Change of** **social impact**
Change of PHQ-9	-			
Change of GAD-7	0.714^**^	-		
Change of health impact	0.285^*^	0.097	-	
Change of social impact	0.440^**^	0.251^*^	0.466^**^	-

PHQ-9, 9-item Patient Health Questionnaire; GAD-7, 7-item Generalized Anxiety Disorder scale;

** P < 0.01;

* *P < 0.05*.

Based on the above results, we included variables that were significantly associated with the improvement of depression and anxiety scores in the linear regression analysis. Results showed that the change of social impact could explain the 12.7% variance of the PHQ-9 improvement and the 10.5% variance of the GAD-7 improvement (PHQ-9: *R*^2^ = 0.127, *t* = 2.691, *P* = 0.009; GAD-7: *R*^2^ = 0.105, *t* = 2.547, *P* = 0.013). Health impact could not explain the variance of improvement in PHQ-9 or GAD-7 (Table 5).

**Table 5:** Results of linear regression analyses on patients' depression and anxiety symptom improvement.

**Variables**	**b**	**SE**	**β**	***t***	***P***	***R*^**2**^**
**Change of PHQ-9**						
Change of social impact	0.560	0.208	0.348	2.691	0.009^**^	0.127
Change of health impact	0.018	0.131	0.018	0.136	0.892	
**Change of GAD-7**						
Change of social impact	0.529	0.208	0.334	2.547	0.013^*^	0.105

b, unstandardized coefficients; β, standardized coefficients;

** p < 0.01;

* *p < 0.05*.

## Discussion

This study explored the mental health recovery and its relationship with negative appraisals in patients who had recovered from COVID-19. We found that 1 month after discharge, patients' depression and anxiety symptoms were significantly improved. The change of the negative appraisals of COVID-19 social impact, rather than the health impact, could explain the depression and anxiety symptom improvement.

Previous studies have shown that patients infected with a novel virus also suffered from mental health problems (24–26). Recently, many studies also revealed that people associated with COVID-19 have suffered varying degrees of psychological distress (9–11, 27–30). However, most of these were cross-sectionally designed and failed to explore the changing process and influencing factors of patients' mental health status. In our study, we found that nearly half of patients with COVID-19 exhibited depression and anxiety symptoms. Although a significant decrease in both depression and anxiety levels was observed, about a third of patients who had recovered from COVID-19 still had obvious psychological distress 1 month after discharge. The improvement in depression and anxiety symptoms was related to the change in the negative appraisals of COVID-19 social impact. The social impact items in the CIS questionnaire (i.e., financial stress, stigma, and concerns about family) did not diminish with the recovery of the disease, yet it explained the change in depression and anxiety levels. Previous studies have shown that greater negative attitudes will lead to a more protracted depressive episode (31, 32). Persistent negative appraisals might lead to long-term depression, while negative emotions may enhance patients' negative appraisals, which is a negative thinking cycle (33). Besides, social support was a main protective factor of maintaining mental wellbeing. However, negative appraisal including fear of infection, feeling of uncertainty, and stigmatization were risk factors for self-isolation which often leads to deprivation of social support (34). Therefore, the negative appraisals, especially those related to COVID-19 social impact, may be one of the sources that perpetuates psychological stress in patients after physical recovery.

It is interesting that the change of negative appraisals related to COVID-19 social impact was more related to symptom improvement in depression and anxiety than the change in negative appraisals of COVID-19 health impact. Several factors might contribute to this phenomenon. On the one hand, COVID-19 had a much lower case fatality rate than that of SARS or Ebola, and most of the patients with COVID-19 presented with mild symptoms. Therefore, the negative appraisals related to COVID-19 health impact might have a limited contribution to depression and anxiety. And the decrease in negative appraisals of COVID-19 health impact was not very closely related to depression and anxiety symptom improvement. On the other hand, COVID-19 is a highly contagious infectious disease and triggers an unprecedented level of panic among the public (35). Patients who recovered from COVID-19 are facing substantial stigma and discrimination when they come back home (36). Although patients have recovered from the infection and finished quarantine for medical observation, they are suffering from social isolation and economic problems or unemployment because of the diagnosis (37). Consistently, a recent study also reported that COVID-19-related financial and social difficulties or concerns were a major risk factor for psychological distress in subjects (38). Therefore, disturbing social problems may be more strongly linked to psychological distress in patients. And the change of negative appraisals related to COVID-19 social impact might contribute to the variation in depression and anxiety. In order to alleviate the depression and anxiety in recovered patients, we could address COVID-19-related social issues. For example, during patients' hospitalization, mental health professionals should be integrated into the medical treatment team to provide targeted psychological interventions. In addition, continued mental health interventions should be prepared for those affected patients after they have been discharged from the designated hospital. These approaches could help to reduce patients' maladaptive beliefs and help to minimize their psychological distress. Except for specific interventions aimed at patients, some effort could be done among the general public and government. For example, health education for the general public could help to reduce the stigma attached to COVID-19. Broadcasting precise and authoritative information such as the fact that recovered patients do not pass the virus to others will help to decrease the discrimination among society (39). In addition, the government could provide unemployment insurance to mitigate the economic impact of COVID-19.

Our study has the following strengths: first, we longitudinally investigated the change in mental distress of patients infected with COVID-19. In addition, we found that the social dimensions played an important role in the mental health of patients during the pandemic. Furthermore, we suggested that more attention should be given to the negative impact of the social dimension on patients' mental health. There are nevertheless several limitations in this study. Firstly, the small sample size and single-site study design limits the ability to generalize the results of the current study. Secondly, online self-reporting was used in the survey, which may bring selection bias since those who cannot use mobile devices were excluded from our study. Thirdly, we recruited 163 participants in the baseline but only 72 participants had complete data at both time points, the response rate was unsatisfactory. However, this may not bring significant bias to the results since we did not detect significant difference in baseline characteristics between the patients with complete data and those excluded. Furthermore, a causal relationship between the change of negative appraisal in COVID-19 social impact and depression or anxiety symptom improvement cannot be drawn from the current study. Long-term change in patients' mental status should be monitored and how the change in negative appraisals influence the future psychological distress should be explored. Finally, using only negative appraisals as a risk factor for the psychological distress in patients was inadequate. Future studies could investigate other influencing factors for patients' mental health during the pandemic.

## Conclusion

Patients who had recovered from COVID-19 showed significant improvement in depression and anxiety symptoms. The change of negative appraisals in the COVID-19 social impact might play a major role in reducing their depression and anxiety. Strategies targeting the reduction of COVID-19-related social issues might be helpful to improve the mental health of recovered patients during the COVID-19 crisis.

## Data Availability Statement

The raw data supporting the conclusions of this article will be made available by the authors, without undue reservation.

## Ethics Statement

The studies involving human participants were reviewed and approved by the ethics review boards of The First Hospital of Changsha. The patients/participants provided their electronic informed consent to participate in this study.

## Author Contributions

All authors had full access to all the data in the study and had full responsibility for the content of the manuscript for publication. KS, JL, and YZha was responsibility for the final review and had final responsibility for the decision to submit for publication.

## Conflict of Interest

The authors declare that the research was conducted in the absence of any commercial or financial relationships that could be construed as a potential conflict of interest.
